# Prevalence of urinary incontinence and associated factors in nursing homes: a multicentre cross-sectional study

**DOI:** 10.1186/s12877-024-04748-1

**Published:** 2024-02-17

**Authors:** Javier Jerez-Roig, Pau Farrés-Godayol, Meltem Yildirim, Anna Escribà-Salvans, Pau Moreno-Martin, Ester Goutan-Roura, Sandra Rierola-Fochs, Montse Romero-Mas, Joanne Booth, Dawn A. Skelton, Maria Giné-Garriga, Eduard Minobes-Molina

**Affiliations:** 1 Research group on Methodology, Methods, Models and Outcomes of Health and Social Sciences (M3O), Faculty of Health Sciences and Welfare, Centre for Health and Social Care Research (CESS), University of Vic-Central University of Catalonia (UVic-UCC), C. Sagrada Família, 7, Barcelona, Vic 08500 Spain; 2 Institute for Research and Innovation in Life Sciences and Health in Central Catalonia (IRIS- CC), Barcelona, Vic Spain; 3 Research group on Tissue Repair and Regeneration Laboratory (TR2Lab), Faculty of Health Sciences and Welfare, Centre for Health and Social Care Research (CESS), University of Vic- Central University of Catalonia (UVic-UCC), Barcelona, Spain; 4 Research Centre for Health (ReaCH), School of Health and Life Sciences, Glasgow Caledonian University, Glasgow, UK; 5 Blanquerna Faculty of Psychology, Education and Sport Sciences, Ramon Llull University, Barcelona, Spain; 6 Blanquerna Faculty of Health Sciences, Ramon Llull University, Barcelona, Spain

**Keywords:** Aged, Nursing homes, Older people, Urinary incontinence, Prevalence, Associated factors

## Abstract

**Background:**

Urinary incontinence (UI) is a common geriatric syndrome with high health and socio-economic impacts in nursing home (NH) residents.

**Objectives:**

To estimate the prevalence and types of UI and its associated factors in older people living in NHs in Central Catalonia (Spain). We also determined the proportion of residents who were receiving behavioural strategies to prevent/manage UI.

**Design and setting:**

Cross-sectional study in 5 NHs conducted from January to March 2020.

**Methods:**

We included consenting residents aged 65 + permanently living in the NHs. Residents who were hospitalized, in a coma or palliative care were excluded. UI was assessed using Section H of the Minimum Data Set. Sociodemographic and health-related variables were examined. Descriptive, bivariate, and multivariate (logistic regression) analyses were performed.

**Results:**

We included 132 subjects (82.6% women), mean age of 85.2 (SD = 7.4) years. The prevalence of UI was 76.5% (95% CI: 68.60-82.93). The most common type was functional UI (45.5%), followed by urgency UI (11.4%). Only 46.2% of residents received at least one behavioural strategy to manage UI. Most sedentary behaviour (SB) variables presented a *p*-value lower than 0.001 in the bivariate analyses, but none remained in the final model. Moderate-severe cognitive impairment (OR = 4.44, *p* =.003), anticholinergic activity (OR = 3.50, *p* =.004) and risk of sarcopenia using SARC-F (OR = 2.75, *p* =.041) were associated with UI.

**Conclusions:**

The prevalence of UI was high in this sample of NH residents compared to the literature, yet less than half received prompted voiding as a strategy to prevent/reduce UI.UI was associated with cognitive impairment, anticholinergic activity, and risk of sarcopenia.

**Supplementary Information:**

The online version contains supplementary material available at 10.1186/s12877-024-04748-1.

## Background

Urinary incontinence (UI) is a common geriatric syndrome affecting bladder health; it affects more than half nursing home (NH) residents, leading to health consequences such as pressure ulcers, urinary tract infections, falls or worsened quality of life [1, 2]. UI represents one of the leading causes of NH admission and a risk of all-cause mortality [3–5]. The causes of UI in frail older adults are multiple and include age-related physiological changes, comorbidity, polypharmacy as well as cognitive and functional impairments [6]. UI leads to a higher burden of health care costs (including costs of labor, laundry, and supplies), and occasionally staff overload and even burnout [2]. In the NH population, UI is strongly associated with cognitive decline, inactivity, immobility, sarcopenia and impairment in activities of daily living performance that could lead to increased sedentary behaviour (SB) [7, 8].

SB is defined as any waking behaviour characterized by an energy expenditure ≤ 1.5 metabolic equivalents (METs), while in a sitting, reclining or lying posture [9]. In older adults, high levels of SB and low levels of awake time movement behaviours (ATMB) are associated with an accelerated aging process and multiple health-related conditions, including frailty, functional decline, osteoporosis, mental disorders, and all-cause mortality [10–17]. NH residents are the least active and accumulate a higher percentage of SB in prolonged and uninterrupted bouts compared to community-dwelling older adults of the same age. NH residents typically spend between 71% and 98% of their waking hours engaged in SB, accumulated in prolonged and uninterrupted periods, which varies based on their level of dependence [18, 19].

An association between urgency UI and SB bouts, defined as the accumulation of uninterrupted and prolonged SB periods, have been found in community-dwelling older women [20]. Emerging research suggests that the duration of SB bouts may have a higher impact on continence than the total amount of time spent in SB throughout the day [20, 21]. However, there are still few studies on this topic, especially in frail older adults and NH residents.

Despite the impact of UI, it remains underdiagnosed and undertreated in this age group: UI is often hidden behind multimorbidity, frailty or other geriatric syndromes, and it also commonly causes embarrassment to sufferers, who avoid admitting their symptoms [22]. Indeed, less than 30% of individuals affected by incontinence in the community seek or receive treatment [23, 24]. Although there are several options available for treating UI, physical (e.g. pelvic floor muscle training) and behavioural (e.g., prompted voiding, bladder training) interventions are recommended by most evidence-based guidelines as first-line approach for treating urgency, stress, and mixed UI [6, 25]. The last Consultation of the International Continence Society states that behavioural strategies with or without exercises to improve mobility and toileting result in modest short-term improvements in UI among NH residents [6]. However, the most frequent management strategy used in the NH setting (and sometimes the only strategy) is the use of absorbent materials [1, 26].

UI is poorly researched in frail older adults [6]. The literature in Spanish NHs is very scarce and published scientific data are not recent [27]. Furthermore, behavioural risk factors such as SB are rarely included in the studies and therefore lifestyle recommendations to prevent or manage UI remain unclear. The Seventh International Consultation on Incontinence states the importance of conducting rigorous studies on frail older adults and lifestyle/behavioural interventions [6]. Therefore, the aim of this study was to estimate the prevalence of UI (and types) as well as analyze its associated factors among older people living in NHs in Central Catalonia (Spain). We also aimed to determine the proportion of residents receiving behavioural strategies to manage UI.

## Methods

### Design

This was a multi-center cross-sectional study (ClinicalTrials register number NCT04297904).

### Settings

The study was conducted from January to March 2020 (until the start of COVID-19 restrictions in Spain) and included data from 5 NHs in Osona county (Barcelona, Spain): 3 subsidized and 2 for-profit NHs. It followed the STROBE (STrengthening the Reporting of OBservational studies in Epidemiology) guidelines [28].

### Sample

Prior to the start of data collection, each NH director agreed to participation in the project with formal consent. The list of residents was then obtained, and the residents were invited to participate in the study according to the eligibility criteria. NH residents (male or female) aged 65 years and over who lived permanently in the institutions and provided informed consent (or his/her legal guardian) was included. Exclusion criteria were residents hospitalized, in a coma or palliative care (prognosis of short life). Full details on the methods are available in the published protocol paper [29].

### Data collection

The research team received training, standard operating procedures and was calibrated to ensure data reliability of the data. The Minimum Data Set (MDS) questionnaire version 3.0, specifically Section H [30] was employed to assess the presence of UI, faecal incontinence (FI), other bladder/bowel conditions, and the implementation of behavioural strategies (e.g., bladder training, scheduled toileting, prompted voiding) during the previous 5 days. Since most residents suffer from cognitive impairment, this information was provided by the staff in charge of direct care of the residents. A trained researcher conducted the interviews and collected the data. In cases where the resident retained cognitive capacity to respond to questionnaires, the continence status was evaluated using the Spanish-validated International Consultation on Incontinence Questionnaire Urinary Incontinence–Short Form (ICIQ UI-SF) [31]. The incontinent group was classified as having any amount of involuntary leakage of urine according to the MDS and/or ICIQ-SF, in line with the definition of the International Continence Society [32]. To obtain the type of UI, the MDS and the ICIQ UI-SF questionnaires were used, and the UI was classified as stress, urgency, mixed, or functional UI. The latter was defined as the loss of urine due to inability or unwillingness to access toilet facilities as a result of physical or cognitive impairment, psychological unwillingness or environmental barriers [27, 33]. As for the ICIQ UI-SF questionnaire, information was obtained through self-reported individual interviews of the residents themselves who maintained sufficient cognitive status to answer questions. For those who did not have the optimal cognitive status to answer them, the information was obtained from the NH staff.

The number of absorbent products (pads/diapers) used daily and nocturia (average number of times waking up to go to the toilet every night during the last 30 days) were also collected by a proxy (NH staff) and double checked using the International Prostate Symptoms Score (IPSS) when the resident had sufficient cognitive capacity [34]. The bowel pattern was assessed through the mean number of voiding times, constipation (fewer than 2 bowel movements per week), diarrhoea and laxatives used during the last 5 days, according to the NH staff [30].

To evaluate SB and time awake movement behaviours (TAMB), the participants wore the ActivPAL3 activity monitor (AP)(PAL Technologies Ltd., Glasgow, UK) for 7 consecutive days following the 24 h protocol during both time awake and sleeping. The device was placed on the anterior medial part of the right thigh, sealed with a flexible nitrile cover, and adhered to the skin with a hypoallergenic adhesive dressing. In cases of stroke, the device was placed on the unaffected leg thigh.

The following sociodemographic information was collected from the NH records or asking the NH staff: age, gender, months of institutionalization, level of education and marital status. The total number of daily medications was registered from the NH records. Active substances within medications were given an Anatomical Therapeutic Chemical(ATC) code [35]. Drugs were grouped according to their first and second level ATC. Anticholinergic activity was calculated according to the Anticholinergic Risk Scale [36]. Weights for each medication (0–3 points) were calculated and then summed to an overall score for each participant. This variable was dichotomized into “no anticholinergic activity” versus “moderate, high and very high”. Chronic conditions included high blood pressure, diabetes, cancer, lung disease, stroke, dementia, Parkinson’s, osteoporosis, kidney failure, dyslipidaemia, cardiac disease and mental illness. We also recorded the number of deliveries (births), history/corrent tobacco use and alcohol intake, urinary tract infections in the last 30 days, bone fractures in the last year and hospitalizations in the last year. Other health-related variables included delirium, ulcers, unintended weight loss in the last year (more than 4.5 kg or more than 5% of previous weight in the last year), number of falls during the last year from NH records, functional capacity (modified Barthel Index, excluding urinary and faecal continence) [37, 38], and frailty (Clinical Frailty Scale) [39], mobility (Rivermead Mobility Index) [40]. According to Prado Villanueva et al. (2011), the following functional capacity categories were considered: independent (80 points), slight dependency (70–79 points), moderate dependency (31–69) and severe dependency (0–30 points) [27].

We used the SARC-F to screen individuals at risk of developing sarcopenia [41]. The consumption of liquids (water and drinks), in millilitres, and types of drinks was collected over a 24-hourperiod, completed by NH staff and the resident themselves where their cognitive capacity was sufficiently preserved. Cognitive status and physical capacity were assessed by trained researchers using the Pfeiffer scale [42] and Short Physical Performance Battery (SPPB) [43], respectively.

### Data analysis

A descriptive analysis was undertaken indicating absolute and relative frequencies for categorical variables as well as mean and standard deviation for quantitative variables. The bivariate analysis, to examine associations, was performed with the Chi-square test (Fisher’s Exact test) or the linear Chi-square test for dichotomous and ordinal variables, respectively. The Kolmogorov-Smirnov test was used to assess the normality of quantitative data. Variables following the normal distribution were analysed using the Student t-test; the Mann Witney test was used for variables not following normal distribution. As an association measure, the odds ratio (OR) was used, considering a confidence level of 95%. All variables with a p-value ≤ 0.20 as well as age and sex were tested with the multivariate analysis following the forward method. Logistic regression was used and the adjustment of the final model was tested with the Hosmer Lemeshow test. A p-value < 0.05 was considered statistically significant. Data were analysed with SPSS version 27 (SPSS Inc., Chicago IL).

### Ethics

Ethical approval was obtained from the Ethics Research Committee of the University of Vic– Central University of Catalonia prior to the commencement of data collection. Signed informed consent was obtained from the residents or their legal guardians.

## Results

**Fig. 1:**
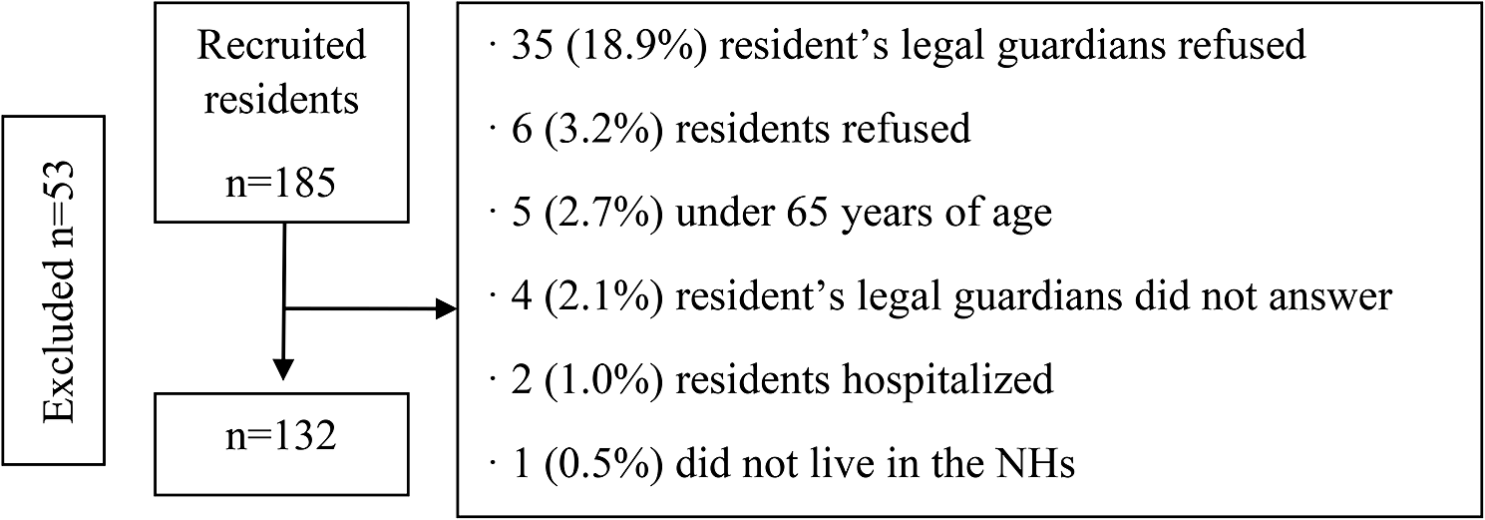
Flowchart of the sampling process of NH residents (Osona, Spain, 2020)

The total sample consisted of 132 residents (82.6% women), with a mean age of 85.2 (SD = 7.4) years. Figure 1 shows the flow chart of the sampling process.

Table 1 shows the sociodemographic and health-related information of the included residents. The average time that residents were in the NH was 38.02 (SD = 44.78) months. From the 132 residents, 76.5% had children and 98.5% residents were diagnosed with at least one condition; mean number of conditions per resident was 5.15 (SD = 2.4). The mean number of medications taken per day was 6.9 (SD = 3.5).

UI was identified in 76.5% (95% CI: 68.6–82.9) residents. The most common type was functional UI (45.4%, 95% CI: 37.2–54.0), followed by urgency UI (11.4, 95% CI: 7.0-17.9). Eight (6.0%) residents reported having UI when the proxy respondent was not aware of urinary losses. Furthermore, 11 (8.3%) residents had UI according to the professional but had not reported experiencing urinary losses themselves. The frequency of dual (urinary and faecal) incontinence was 28.8% (95% CI: 21.8–37.0). Only 2 (1.5%) residents suffered from faecal incontinence but not UI.

The mean number of absorbent products used per day across the whole sample was 3.0 (SD = 3.1), with the incontinent group using3.9 (SD = 3.1) and the continent group using 0.4 (SD = 0.8). Almost half (46.2%, 95% CI: 37.9–54.7%) of the residents received a behavioural strategy to prevent or manage their UI, prompted voiding being the applied method in all cases. Total or partial improvement of the continence status was obtained in more than half (57.4%) of the cases who received this strategy, according to the NH staff perspectives. The total average fluid consumption was 1828.5 (SD = 752.9) millilitres (mL) per day: 16.8 (SD = 66.9) mL of cold drinks with caffeine, 206.9 (SD = 298.4) mL of hot drinks with caffeine, 1419.4 (SD = 611.2) mL of non-caffeine cold drinks, 172.7 (SD = 197.8) mL of non-caffeine hot drinks, and 5.0 (SD = 39.6) mL of alcohol. For additional sociodemographic and health-related information on the sample, refer to Table A1 in the Annexes.

**Table 1:** Sociodemographic and health-related information of the sample of NH residents (*n* = 132) from Osona, Spain (2020)

	n	Frequency (%)
Education level		
Illiterate / no schooling	38	28.8
Primary school	50	37.9
High school	7	5.3
College education	4	3.0
Unknown	33	25.9
Marital status		
Single	17	12.9
Married/partner	19	14.4
Divorced	3	2.3
Widowed	76	57.6
Unknown	17	12.9
Type of NH		
Public	23	17.4
State-subsidised places	64	48.5
Private	45	34.1
Diagnosed conditions		
High blood pressure	83	62.9
Dementia	71	53.8
Cardiac disease	53	40.2
Dyslipidaemia	41	31.1
Kidney failure	36	27.3
Diabetes	36	27.3
Depression	36	27.3
Urinary continence		
Continent	39	29.5
Occasionally incontinent	44	33.3
Frequently incontinent	21	15.9
Always incontinent	26	19.7
Catheter	2	1.5
UI types (*n* = 101)		
Functional	60	45.5
Urgency	15	11.4
Stress	4	3.0
Mixed	11	8.3
Undetermined	11	8.3
Behavioural strategy to prevent/manage UI		
No	71	53.8
Yes	61	46.2
Response to programme		
No improvement	25	41.0
Partial improvement	21	34.4
Total improvement (continence)	14	23.0
Undetermined	1	16.4
UI duration (*n* = 101)		
<1 month	2	1.9
1 month– 1 year	9	8.9
> 1 year	70	69.3
Undetermined	20	19.8
UI– amount of leakages (*n* = 101)		
Small (drops)	77	76.2
Large	13	12.8
Undetermined	11	10.8
UI– pattern (*n* = 101)		
Day	1	0.9
Night	28	27.7
Day and night	59	58.4
Undetermined	13	12.9
Residents with bowel Health issues		
Fecal incontinence	40	30.3
Diarrhoea	14	10.6
Laxatives	47	35.6
Constipation	25	18.9
Frailty (Clinical Frailty Scale)		
Very fit	1	0.8
Well	15	11.4
Managing Well	5	3.8
Vulnerable	6	4.6
Mildly Frail	22	16.7
Moderately Frail	39	29.6
Severely Frail	33	25.0
Very Severely Frail	11	8.3
Terminally ill	0	0
Cognitive capacity (Pfeiffer)		
Intact	26	19.7
Slight impairment	16	12.1
Moderate impairment	29	22.0
Severe impairment	56	42.4
Unknown	5	3.8
ADL limitations (Barthel)		
Independent	7	5.3
Slight dependency	20	15.2
Moderate dependency	52	39.4
Severe dependency	53	40.2
Nutritional state (Mini Nutritional Assessment)		
Normal nutritional status	31	23.5
At risk of malnutrition	70	53.0
Malnourished	9	6.8
Unknown	22	16.7

Key: UI = Urinary incontinence

Table 2 includes bivariate analysis to consider the associations between UI and quantitative independent variables. All the variables except age, number of SB bouts > 60 min and absolute time in SB presented a *p*-value of 0.001 or lower. Results from bivariate analysis between UI and categorical variables are shown in Table 3. UI was significantly associated with diagnosed dementia, depression, visual deficit, digestive disease, group S drugs, anticholinergic medication, nocturia, risk of sarcopenia, physical performance, cognitive impairment, malnutrition, frailty, ADL limitations and faecal incontinence.

**Table 2:** Unadjusted associations of UI and independent quantitative variables in NH residents from Osona, Spain

Variables	Continent (mean and SD) *n* = 24	Incontinent (mean and SD) *n* = 71	Mean difference (95% CI)	*p* value
Age	83.84(6.67)	85.59(7.59)	-1.751 (-4.59-1.09)	0.211
Hours awake (h)	13.82 (1.03)	12.00(1.83)	1.82 (1.03–2.60)	< 0.001
Standing duration (h)	3.29 (2.23)	1.27 (1.74)	2.02 (0.50-1.00)	< 0.001
% time awake standing	23.59 (15.32)	9.83 (12.72)	13.76 (6.70-20.81)	< 0.001
Walking duration (h)	0.81 (0.62)	0.29 (0.46)	0.51 (0.23–0.80)	< 0.001
% time awake walking	5.92 (4.74)	2.30 (4.01)	3.61 (1.42–5.80)	< 0.001
Absolute time upright (h)	4.10 (2.39)	1.56 (2.08)	2.54 (1.43–3.65)	< 0.001
% time awake upright	29.51 (16.64)	12.14 (15.70)	17.36 (9.51–25.21)	< 0.001
Sit to stand transitions	33.71 (11.63)	20.46 (21.14)	13.24 (4.22–22.26)	< 0.001
Absolute time in SB (h)	9.71 (2.33)	10.44 (2.00)	-0.72 (-1.70-0.25)	0.184
% time awake in SB	70.48 (16.64)	87.85 (15.70)	-17.36 (-25.21–9.51)	< 0.001
Number of SB bouts < 30 min	27.21 (12.00)	16.16 (20.13)	11.05 (4.22–17.88)	< 0.001
Absolute time spent in bouts < 30 min (h)	2.53 (1.26)	1.45 (1.66)	1.07 (0.33–1.81)	< 0.001
% time awake in bouts < 30 min	18.43 (9.54)	11.27 (12.37)	7.16 (1.65–12.66)	0.001
Number of SB bouts between 30–60 min	3.92 (1.66)	1.76 (2.15)	2.15 (1.19–3.11)	< 0.001
Absolute time spent in bouts between 30–60 min (h)	2.78 (1.22)	1.23 (1.44)	1.54 (0.93–2.15)	< 0.001
% time awake in bouts between 30–60 min	19.97 (8.60	9.53 (10.51)	10.43 (5.71–15.16)	< 0.001
Number of SB bouts > 60 min	2.50 (1.14)	2.59 (1.05)	-0.88 (-0.62–0.44)	0.589
Absolute time spent in bouts > 60 min (h)	4.40 (3.03)	7.75 (3.76)	-3.34 (-5.03–1.65)	< 0.001
% of time awake in bouts > 60 min	32.07 (23.00)	67.04 (33.64)	-34.96 (-49.66–20.26)	< 0.001
Average duration of SB bouts (min)	22.44 (17.71)	93.07 (102.61)	-70.62 (-122.57–28.67)	< 0.001

Key: SB = sedentary behaviour; ATMB = awake time movement behaviour; SD = standard deviation; h = hours; min = minutes; %=percentage

**Table 3:** Unadjusted associations of UI and categorical variables with p value lower than 0.20 in NH residents from Osona, Spain

UI				
	Yes n (%) *n* = 101	No n (%) *n* = 31	*p* value	OR (95% CI)
Sex				
Men	14 (13.9)	9 (29.0)		reference
Women	87 (86.1)	22 (71.0)	0.051	2.54 (0.97-6.63)
Primary school completed				
Yes	48 (78.7)	13 (21.3)		reference
No	35 (92.1)	3 (7.9)	0.078	3.16 (0.83-11.93)
Dementia				
No	39 (66.1)	20 (33.9)		reference
Yes	60 (84.5)	11 (15.5)	0.014	2.79 (1.20–6.47)
Stroke				
No	76 (73.1)	28 (26.9)		reference
Yes	23 (88.5)	3 (11.5)	0.100	2.82 (0.78-10.14)
Depression				
No	67 (71.3)	27 (28.7)		reference
Yes	32 (88.9)	4 (11.1)	0.035	3.22 (1.04–9.99)
Osteoarthritis				
No	88 (78.6%)	24 (21.4%)		reference
Yes	11 (61.1%)	7 (38.9%)	.136ª	0.42 (0.15-1.22)
Digestive disease				
No	87 (80.6)	21 (19.4)		reference
Yes	12 (54.5	10 (45.5	0.014	0.29 (0.11-0.76)
Group C drugs				
No	49 (84.5)	9 (15.5)		reference
1 or more	51 (70.8)	21 (29.2)	0.066	0.44 (0.18-1.06)
Group S drugs				
No	97 (78.9)	26 (21.1)		reference
1 or more	3 (42.9)	4 (21.1)	.049ª	0.20 (0.42-0.95)
Drugs to reduce micturition				
No	8 (61.5)	5 (38.5)		reference
1 or more	92 (78.6)	25 (21.4)	.176ª	2.30 (0.69-7.64)
Anticholinergic activity				
No	30 (62.5)	18 (37.5%)		reference
Yes (moderate-very high)	70 (85.4)	12 (14.6)	0.005	3.50 (1.50–8.16)
Falls last year				
No	51 (71.8)	20 (28.2)		reference
Yes	50 (82)	1 (18.0)	0.171	1.78 (0.77-4.09)
Nocturia				
No	55 (90.2)	6 (9.8)		reference
Yes	35 (71.4	14 (28.6)	0.011	0.27 (0.09-0.77)
Risk of sarcopenia (SARC-F)				
No	18 (58.1)	13 (41.9)		reference
Yes	82 (82.0)	18 (18.0)	0.006	3.29 (1.36–7.90)
Physical function (SPPB)				
Robust/Prefrailty/Frail	26 (50)	26 (50)		reference
Disability	69 (94.5)	4 (5.5)	< 0.001	17.25 (5.48–54.21)
Mini Nutritional Assessment (MNA)				
Normal	20 (64.5)	11 (35.5)		reference
At risk– Malnourished	66 (83.5)	13 (16.5)	0.030	2.7 (1.08–7.19)
Cognitive capacity (Pfeiffer questionnaire)				
Normal/Slight	24 (57.1)	18 (42.9)		reference
Moderate/Severe	77 (85.6)	13 (14.4)	< 0.001	4.44 (1.90-10.37)
ADL Limitations (Barthel)				
Independent/Slight dependency	10 (37.0)	17 (63.0)		reference
Moderate/Severe dependency	91 (86.7)	14 (13.3)	< 0.001	11.05 (4.22–28.93)
Fecal Incontinence				
No	63 (68.5)	29 (31.5)		reference
Yes	38 (95.0)	2 (5.0)	< 0.001	8.74 (1.97–38.74)

Keynotes: CI = confidence interval; OR = Odds Ratio; ª Fisher’s Exact Test

Table 4 shows the final model as a result of logistic regression, showing data from bivariate analysis of the three variables who remained in the multivariate analysis (cognitive impairment, anticholinergic activity and risk of sarcopenia).

**Table 4:** Multivariate final model analysis of the sample of NH residents

	UI							
	Yes		No					
	n	%	n	%	*p* value	OR (CI:95%)	*p* value	Adjusted OR (CI:95%)
Cognitive capacity								
Normal/Slight	24	57.1	18	42.9				reference
Moderate/ Severe	77	85.6	13	14.4	< 0.001	4.44 (1.90-10.37)	0.003	4.25 (1.66–10.91)
Anticholinergic activity								
No	30	62.5	18	37.56	30			reference
Yes (moderate/very high)	70	85.4	12	14.65	0.005	3.50 (1.50–8.16)	0.004	4.01 (1.57–10.23)
Risk of Sarcopenia (SARC-F)								
No	18	58.1	13	41.9	18			reference
Yes	82	82.0	18	18.09	0.006	3.29 (1.36–7.90)	0.041	2.75 (1.04–7.30)

Key: CI = confidence interval; OR = Odds Ratio

## Discussion

This work aimed to estimate the prevalence and types of UI, associated factors and the behavioural strategies used to manage this geriatric syndrome in NHs from Osona (Central Catalonia, Spain). According to the MDS, 76.5% of older adults suffered some type of urinary losses, with functional UI being the most frequent type, followed by urgency UI. Most incontinent residents had UI of long duration (more than 1 year), the amount of urinary leaked was predominantly low (drops) and followed a night or day-night pattern. Only 46% residents received behavioural interventions designed to prevent or actively manage UI; for all cases this was prompted voiding. Cognitive impairment, anticholinergic activity and risk of sarcopenia represented factors associated with UI in this sample of NH residents.

The prevalence of UI found in our study is at the higher end of the spectrum reported in the literature, which generally ranges from 13 to 77% [2]. It is also substantially higher than the prevalence (53.6%) in another Spanish study [27], and the recently published comparison of UI prevalence in NHs in Austria (35.1%), Netherlands (27.9%), UK (18.4%) and Turkey (13.8%) [27, 44]. Residents with UI in our study were frail (83%), cognitively impaired (81%), sarcopenic (77%), malnourished (79%), dependent in ADLS (80% moderate to severe on Barthel) and disabled (89% on SPPB). In Catalonia (Spain)the profile of NH residents tends to be increasingly older and frailer, which could explain the higher prevalence of UI found in our sample [45].

The most common type was functional UI, followed by urgency and mixed UI. The frequency of urgency UI found in our study (11%) is in line with that reported in other studies, ranging from8 to 15% [1, 27]. Urgency and mixed UI increase with ageing, but in NH residents, who represent the frailest segment of the population, significant cognitive and physical difficulties reaching and using a toilet mean that functional UI is especially prevalent [1, 45]. This explains the high proportion of residents with functional UI found in our study (45%), higher than reported in previous literature (20–33%) [1, 27]. However, the impossibility of interviewing all residents (due to cognitive impairment) and possible inaccuracies in information provided by proxy respondents, should be recognised as a limitation. Indeed, there were 19 cases (representing approximately 14% of the sample) of mismatch in the continence status between the information provided by the NH staff and the resident. Some proportion of mismatch in diagnosis is common in research on UI; some older adults feel uncomfortable or ashamed when interviewed and can deny their incontinence status [46, 47]. On the other hand, NH staff sometimes may not know the continence status of all their most independent residents. Additionally, the assessment of UI types is not routine in NHs and staff frequently lack knowledge on incontinence [29, 48].

Passive containment using absorbent pads is the most common conservative strategy to manage UI in NHs and this has been confirmed in our study as 96% incontinent residents used absorbent products [44]. Only 46% of the sample received any type of active behavioural management, all of these received prompted voiding, an appropriate intervention for this frail NH sample and the type of UI observed. The latter is a structured toileting program suitable for adults with impaired cognitive function and all types of UI, comprising scheduled voiding, based on recognition by the resident of their need to void, following prompting by a caregiver. The use of prompted voiding in NHs ranges from 8 to 12% in developing countries such as Brazil and Turkey to 77% in the UK [1, 44]. It is worth noting that evidence suggests prompted voiding reduces incontinence by 9–43%, but it requires training, time and staff commitment and coordination [6]. Indeed, the proxy respondents in our study reported that the application of prompted voiding led to a total or partial improvement in continence in 57.4% of residents.

The strongest factor associated with UI in our study was cognitive impairment, one of the most frequently reported in the literature [2, 49]; older adults with moderate-severe cognitive impairment were 4.5-fold more likely to have UI than those with no or mild cognitive impairment. Descriptive data from this sample (53.8% with diagnosed dementia; 42.4% with severe cognitive impairment according to the Pfeiffer test) indicates that most residents presented with altered cognition. UI is a multicausal condition [50] in which cognitive factors play an important, however, still unexplained role. The relationship between UI and cognition was explored by Hatta et al. (2011) [51] who suggest the peripheral nerves involved in urinary bladder function are controlled by urination centres in the brain stem and the prefrontal cortex. The prefrontal cortex is one area known to be associated with urinary symptoms of urgency and frequency [52]. Any damage to the brain can affect neural control of the bladder and manifest as urgency and frequency (overactive bladder) which in advancing dementia disease may directly cause functional UI. This may explain the strong association of UI with neurological conditions such as stroke [53]. Furthermore, cognitive impairment and dementia are also associated with impaired ability to communicate the need to void or successfully navigate to and use the toilet [50]. These may be indirect explanatory mechanisms for the strong association no observed in this frail population.

Drugs with anticholinergic activity are used for the treatment of many prevalent conditions in NHs such as mental diseases, respiratory disorders or even overactive bladder, a common cause of UI in NHs [54]. Urgency UI and overactive bladder are sometimes treated with anticholinergic drugs such as solifenacin, tolterodin or trospium (group G in the ATC classification). However, only 6% of incontinent residents in our study took this type of drugs for the urinary tract. Therefore, our main hypothesis to explain the association with UI relates to adverse effects associated with anticholinergic activity which affect up to 87% of NH residents [54]. Anticholinergics can impair emptying and cause constipation and urinary retention [6]. The use of anticholinergic medications has been also associated with cognitive decline and decrease effective toileting ability, which in turn may lead to continence decline [55].

Another factor associated with UI in our study was risk of sarcopenia, part of the frailty syndrome, together with cognitive impairment. The older residents in this study were frail and dependent in ADL, aligning with other studies where NH residents with UI have increased odds of functional problems/dependence compared to continent residents. Sarcopenia may explain the relationship between frailty, ADL limitations and UI, as it may result in weakened pelvic floor muscles and/or impaired ability to independently toilet, leading to functional UI [56].

Several investigations support this relationship [57–60]. Moreover, a recent study [61] has concluded that UI was strongly associated with musculoskeletal impairments in functioning and mobility in older people. There is a correlation between measured muscle strength with the handgrip test, which is one of the diagnostic criteria for sarcopenia, and the perineometer test, which measures UI. Therefore, low muscle strength may be a marker of pelvic floor muscle weakness leading to UI [62].

Whilst studies have objectively measured SB in NHs [19] none have considered its associations with UI. In this study objectively measured SB has shown to be associated in bivariate analysis with total UI. The residents in this study had similar percentage of waking time in SB as a recent review. However, those with incontinence were sedentary for longer than those without UI (87% vs. 70%) and walked for less time daily (0.2 h vs. 0.8 h). Residents with UI had half the number of sit to stand transfers (20 vs. 33) and were sedentary for longer bout durations (22 vs. 93 min). There are few published interventions aimed at reducing SB in residents of NHs [63, 64]; but one study, in frailer older people living in sheltered housing, has shown that essentially breaking up prolonged bouts of SB with standing, for 30 s approximately hourly [65], leads to increased physical function (Timed up and go and 30 s chair rise) which may help in maintenance of functional continence.

The main limitation of this study was its relatively small sample size. Data collection was stopped due to the covid-19 pandemic in March 2020 with consequent restrictions lasting several months. Despite exceeding the sample size calculation, SB variables presented with more than 20% missing data(for different reasons such as refusals), limiting its use in multivariate analysis.One aspect that may influence the prevalence data is that 21% of legal guardians refused to participate in the study, a high figure compared to previous studies (0–18%) [1, 33, 44]. There is still relatively little research in this area in comparison to research on ageing in general [66]. This may be partly explained by the complexities associated with recruiting vulnerable people and genuine ethical concerns about involving this group in research in the eyes of family members [67]. However, we were still able to apply a comprehensive assessment of residents from 5 NHs, including a high number of variables. Studies are necessary to further explore the role of activity patterns on pelvic health in older people. Another limitation is that the cross-sectional design of the study cannot provide evidence on the cause-effect relationship between the dependent and independent variables.

## Conclusions

Approximately 3 out of 4 NH residents in this sample presented with some degree of UI, with the functional type (due to cognitive/physical restraints) the most common form. Prompted voiding was used in almost half of the sample. Cognitive impairment, anticholinergic activity and risk of sarcopenia were factors associated with UI. These results highlight the importance of reviewing residents’ medications to reduce anticholinergic burden, as well as applying mobility and behavioural strategies to promote effective toileting.

### Electronic supplementary material

Below is the link to the electronic supplementary material.

**Supplementary Material 1**: Appendix A. Table A1. Additional sociodemographic and health-related information of the sample of NH residents (n=132) from Osona, Spain (2020). Table A2. Bivariate analysis between UI and categorical variables (with p value higher than 0.20) in NH residents from Osona, Spain (2020).

## Data Availability

The datasets used and/or analysed during this study are available from the corresponding author on reasonable request.
