# Supplementary material for: Prevalence of urinary incontinence and associated factors in nursing homes: a multicentre cross-sectional study
Source: BMC Geriatr. 2024 Feb 17;24:169. doi: 10.1186/s12877-024-04748-1 (PMC10874568; doi:10.1186/s12877-024-04748-1)
Supplement: Supplementary file 1 — Supplementary Material 1: Appendix A. Table A1. Additional sociodemographic and health-related information of the sample of NH residents (n=132) from Osona, Spain (2020). Table A2. Bivariate analysis between UI and categorical variables (with p value higher than 0.20) in NH residents from Osona, Spain (2020). [file 12877_2024_4748_MOESM1_ESM.docx]

**Appendix A**

| **Table A1.** Additional sociodemographic and health-related information of the sample of NH residents (n=132) from Osona, Spain (2020). | | | | |
| --- | --- | --- | --- | --- |
|  |  |  | **n** | **Frequency (%)** |
| Alcohol consumption | | | | |
| No | | | 62 | 47.0 |
| Former drinker | | | 8 | 6.1 |
| Yes | | | 3 | 2.3 |
| Unknown | | | 59 | 44.7 |
| Diagnosed conditions | | |  |  |
| Stroke | | | 26 | 19.7 |
| Cancer | | | 25 | 18.9 |
| Lung disease | | | 24 | 18.2 |
| Digestive disease | | | 22 | 16.7 |
| Osteoporosis | | | 22 | 16.7 |
| Mental disorder | | | 21 | 15.9 |
| Falls in the last year | | | 61 | 46.2 |
| Episodes of delirium in the last year | | | 6 | 4.5 |
| Lost weight in the last 12 months | | | 25 | 18.9 |
| Ulcers | | | 6 | 4.5 |
| Hospitalized in the last 12 months | | |  |  |
| No | | | 75 | 56.8 |
| Yes | | | 18 | 13.6 |
| Unknown | | | 39 | 29.5 |
| Urinary tract infection in the last 30 days | | |  |  |
| No | | | 116 | 87.9 |
| Yes | | | 11 | 8.3 |
| Unknown | | | 5 | 3.8 |
| Medication (ATC Classification) ^a^ | | |  |  |
| Group A | | | 86 | 65.2 |
| Group B | | | 57 | 43.2 |
| Group C | | | 72 | 54.5 |
| Group D | | | 6 | 4.5 |
| Group G | | | 10 | 7.6 |
| Group H | | | 20 | 15.2 |
| Group J | | | 5 | 3.8 |
| Group L | | | 2 | 1.5 |
| Group M | | | 10 | 7.6 |
| Group N | | | 127 | 96.2 |
| Group R | | | 30 | 22.7 |
| Group S | | | 7 | 5.3 |
| Group V | | | 1 | 0.8 |
| Body Mass Index (BMI) | | |  |  |
| Under weight | | | 45 | 34.1 |
| Normal/Overweight | | | 26 | 19.7 |
| Obese | | | 21 | 15.9 |
| Unknown | | | 40 | 30.3 |
| Physical function (SPPB) | | |  |  |
| Robustness | | | 5 | 3.8 |
| Prefrailty | | | 18 | 13.6 |
| Frailty | | | 29 | 22.0 |
| Disability | | | 73 | 55.3 |
| Unknown | | | 7 | 5.3 |
| Nutritional state (Mini Nutritional Assessment) | | |  |  |
| Normal nutritional status | | | 31 | 23.5 |
| At risk of malnutrition | | | 70 | 53.0 |
| Malnourished | | | 9 | 6.8 |
| Unknown | | | 22 | 16.7 |
| Key: ^a^ Drugs: N (Nervous System), A (Alimentary tract and metabolism), C (Cardiovascular system), B (Blood and blood forming organs), R (Respiratory System), H (Systemic hormonal preparations, excl. Sex hormones and insulins), G (Genito urinary System/sex hormones), M (Musculo-skeletal system), S (Ophthalmological), J (Anti-infective), D (Dermatological), L (Antineoplastic agents) and V (Immunomodulating agents). | | | | |

| **Table A2.** Bivariate analysis between UI and categorical variables (with *p* value higher than 0.20) in NH residents from Osona, Spain (2020). | | | | |
| --- | --- | --- | --- | --- |
| **UI** | | | | |
|  | **Yes**  **n (%)** | **No**  **n (%)** | ***p* value** | **OR (CI:95%)** |
| Group G drugs | | | | |
| No | 94 (78.3%) | 26 (21.7%) |  | reference |
| 1 or more | 6 (60.0%) | 4 (40.0%) | 0.238ª | 0.41 (0.10-1.58) |
| Group H drugs | | | | |
| No | 86 (78.2%) | 24 (21.8%) |  | reference |
| 1 or more | 14 (70.0%) | 6 (30.0%) | 0.403ª | 0.65 (0.22-1.87) |
| Group J drugs | | | | |
| No | 97 (77.6%) | 28 (22.4%) |  | reference |
| 1 or more | 3 (60.0%) | 2 (40.0%) | 0.326ª | 0.43 (0.69-2.72) |
| Group L drugs | | | | |
| No | 98 (76.6%) | 30 (23.4%) |  | reference |
| 1 or more | 2 (100.0% | 0 (0%) | 1.000ª | 0.31 (0.07-1.23) |
| Group R drugs | | | | |
| No | 77 (77.0%) | 23 (23.0%) |  | reference |
| 1 or more | 23 (76.7%) | 7 (23.3%) | 0.970ª | 0.98 (0.37-2.57) |
| Group V drugs | | | | |
| No | 99 (76.7%) | 30 (23.3%) |  | reference |
| 1 or more | 1 (100.0%) | 0 (0%) | 1.000ª | 0.47 (0.17-1.32) |
| Group B drugs | | | | |
| No | 56 (76.7%) | 17 (23.3%) |  | reference |
| 1 or more | 44 (77.2%) | 13 (22.8%) | 0.949ª | 1.02 (0.45-2.34) |
| Group D drugs | | | | |
| No | 96 (77.4%) | 28 (22.6%) |  | reference |
| 1 or more | 4 (66.7%) | 2 (33.3%) | 0.621ª | 0.58 (0.10-3.35) |
| Group N drugs | | | | |
| No | 2 (66.7%) | 1 (33.3%) |  | reference |
| 1 or more | 98 (77.2%) | 29 (22.8%) | 0.548ª | 0.43 (0.16-1.16) |
| High blood pressure | | | | |
| No | 33 (70.2%) | 14 (29.8%) |  | reference |
| Yes | 66 (79.5%) | 17 (20.5%) | 0.232 | 1.64 (0.72-3.74) |
| Diabetes | | | | |
| No | 70 (74.5%) | 24 (25.5%) |  | reference |
| Yes | 29 (80.6%) | 7 (19.4%) | 0.466 | 1.42 (0.55-3.63) |
| Lung disease | | | | |
| No | 80 (75.5%) | 26 (24.5%) |  | reference |
| Yes | 19 (79.2%) | 5 (20.8%) | 0.701 | 1.23 (0.41-3.63) |
| Dyslipidaemia |  |  |  |  |
| No | 68 (76.4%) | 21 (23.6%) |  | reference |
| Yes | 31 (75.6%) | 10 (24.4%) | 0.921 | 0.95 (0.40-2.27) |
| Kidney failure | | | | |
| No | 73 (77.7%) | 21 (22.3%) |  | reference |
| Yes | 26 (72.2%) | 10 (27.8%) | 0.501 | 0.74 (0.31-1.79) |
| Cardiac disease | | | | |
| No | 57 (74.0%) | 20 (26.0%) |  | reference |
| Yes | 42 (79.2%) | 11 (20.8%) | 0.471 | 1.34 (0.58-3.09) |
| Osteoporosis | | | | |
| No | 82 (75.9%) | 26 (24.1%) |  | reference |
| Yes | 17 (77.3%) | 5 (22.7%) | 0.893 | 1.07 (0.36-3.20) |
| Mental disorder | | | | |
| No | 82 (75.2%) | 27 (24.8%) |  | reference |
| Yes | 17 (81.0%) | 4 (19.0%) | 0.573 | 1.39 (0.43-4.52) |
| Key: ªFisher’s Exact Test. | | | | |
